# The Cadherin Cry1Ac Binding-Region is Necessary for the Cooperative Effect with ABCC2 Transporter Enhancing Insecticidal Activity of *Bacillus thuringiensis* Cry1Ac Toxin

**DOI:** 10.3390/toxins11090538

**Published:** 2019-09-14

**Authors:** Yuemin Ma, Jianfeng Zhang, Yutao Xiao, Yanchao Yang, Chenxi Liu, Rong Peng, Yongbo Yang, Alejandra Bravo, Mario Soberón, Kaiyu Liu

**Affiliations:** 1 Institute of Entomology, School of Life Sciences, Central China Normal University, Wuhan 430079, China; ymma@mails.ccnu.edu.cn (Y.M.); jianfengzhang@mails.ccnu.edu.cn (J.Z.); yangyanchao@mails.ccnu.edu.cn (Y.Y.); pengrong@mail.ccnu.edu.cn (R.P.); yongboyang@mail.ccnu.edu.cn (Y.Y.); 2 Agricultural Genomics Institute at Shenzhen, Chinese Academy of Agricultural Sciences, Shenzhen 518120, China; xiaoyutao@caas.cn; 3 State Key Laboratory for Biology of Plant Disease and Insect Pests, Chinese Academy of Agricultural Sciences, West Yuanmingyuan Road, Beijing 100193, China; liuchenxi@caas.cn; 4 Instituto de Biotecnología, Universidad Nacional Autónoma de México, Apdo. Postal 510-3, Cuernavaca 62250, Morelos, Mexico; bravo@ibt.unam.mx (A.B.);

**Keywords:** *Helicoverpa armigera*, *Spodoptera litura*, cadherin, ABCC2 transporter, *Bacillus thuringiensis*, synergism, Cry1Ac

## Abstract

*Bacillus thuringiensis* Cry1Ac toxin binds to midgut proteins, as cadherin (CAD) and ABCC2 transporter, to form pores leading to larval death. In cell lines, co-expression of CAD and ABCC2 enhance Cry1Ac toxicity significantly, but the mechanism remains elusive. Here, we show that the expression of *Helicoverpa armigera* CAD (HaCAD-GFP) in Hi5 cells induces susceptibility to Cry1Ac and enhanced Cry1Ac toxicity when co-expressed with *H. armigera* ABCC2 (HaABCC2-GFP), since Cry1Ac toxicity increased 735-fold compared to Hi5 cells expressing HaCAD-GFP alone or 28-fold compared to HaABCC2-GFP alone. In contrast, the expression of the *Spodoptera litura* CAD (SlCAD-GFP) in Hi5 cells did not induce susceptibility to Cry1Ac nor it potentiated Cry1Ac toxicity with HaABCC2-GFP. To identify the CAD regions involved in the enhancement of Cry1Ac toxicity with ABCC2, the different CAD domains were replaced between SlCAD-GFP and HaCad-GFP proteins, and cytotoxicity assays were performed in Hi5 cells in the absence or presence of HaABCC2-GFP. The HaCAD toxin-binding region (TB), specifically the CAD repeat-11, was necessary to enhance Cry1Ac toxicity with ABCC2. We propose that CAD TB is involved in recruiting Cry1Ac to localize it in a good position for its interaction with the ABCC2, resulting in efficient toxin membrane insertion enhancing Cry1Ac toxicity.

## 1. Introduction

*Bacillus thuringiensis* (Bt) produces different Cry toxins that have been extensively used in spray formulations for insect control. Also, the expression of certain *cry* genes in different crops plants such as corn, cotton, and soya have resulted in an efficient protection of these Bt-crops from insect attack [1,2]. The Cry proteins are highly specific since they interact with different receptors such as aminopeptidase N (APN), alkaline phosphatase (ALP), cadherin (CAD) and an ATP-binding cassette transporter proteins (ABCC2) which are located in the apical membrane of the insect midgut cells [3,4,5]. Cry toxin receptors play important roles in toxin binding, inducing toxin oligomerization, insertion into the membrane and pore formation leading to lysis of the insect midgut cells and larval death [6,7,8]. It has been shown that CAD facilitates the formation of a pre-pore oligomer, while ALP and APN are involved in oligomer membrane insertion [6,9,10]. In the case of ABCC2, it was suggested that binding of Cry1Ac to ABCC2 fulfills both roles, oligomerization of the toxin and insertion of the oligomers into the membrane of larval midgut cells [7,11]. Interestingly, it has been shown that co-expression of CAD and ABCC2 from *Bombyx mori* in Sf9 cells have a potentiation effect on Cry1Ac toxicity resulting in 100 fold higher toxicity in the presence of both receptors, than in cell expressing only BmABCC2 [11,12]. Other examples of similar enhancement of Cry1 toxicity due to the presence of both CAD and ABCC2 receptors from other lepidopteran insect species have also been reported [13,14,15].

The CAD receptor is composed of three domains, the extracellular domain, the transmembrane domain (TM) and a cytoplasmic domain (CPD). The extracellular domain consists of a membrane proximal region (MP) and 11 to 12 cadherin repeats (CR) that participate in Ca^2+^-binding. It has been shown that the CR10-11 of *Helicoverpa armigera* CAD (HaCAD) contains the toxin-binding region (TB) that interacts with Cry1Ac toxin [3,16]. The role of CAD domains in Cry1A toxicity has been studied through site directed mutagenesis including the truncation or deletion of some domains of the protein [17,18,19,20]. The CAD TB plays an important role in mediating toxicity of Cry toxins by facilitating toxin oligomerization [6,16,21]. The other CR regions are not necessary for Cry toxicity. For example, a HaCAD mutant, where the first nine CR were deleted, was still able to confer Cry1Ac toxicity to Sf9 cells similar to the complete HaCAD protein [18]. Regarding the MP region, there is evidence that it is not involved in Cry1Ac toxicity, since CAD fragments containing only the TB domain or CR12 from *Manduca sexta* CAD (MsCAD) were still able to synergize toxicity of Cry1Ac in different insect larvae [22,23]. Finally, the role of CPD is controversial, it was reported that removal of this region in HaCAD resulted in slight but significant loss of Cry1Ac susceptibility in Sf9 cells compared to the complete HaCAD [18]. However, the results with *B. mori* CAD (BmCAD) were different, since a deletion of this region still was able to confer susceptibility to Cry1Aa and Cry1Ab in Sf9 cells and to enhance Cry1Ac toxicity with ABCC2 similar to the complete BmCAD [24].

To identify the specific CAD regions involved in the cooperative effect with ABCC2 to potentiate Cry1Ac toxicity, different hybrid CADs were constructed and characterized. We used two CAD proteins, the HaCAD-GFP that was able to mediate cytotoxicity of Cry1Ac to Hi5 cells and the *Spodoptera litura* CAD (SlCAD-GFP) that did not induce susceptibility of Hi5 cells to Cry1Ac toxin. The HaCAD-GFP showed a strong potentiation effect in Cry1Ac toxicity with ABCC2 from *H. armigera* (HaABCC2-GFP) when both proteins were co-expressed in Hi5 cells and the SlCAD-GFP did not show this potentiation effect with HaABCC2-GFP. Our data allowed us to propose a model that could explain the initial steps in the synergism between CAD and ABCC2 proteins inducing high levels of Cry1Ac toxicity.

## 2. Results

### 2.1. HaCAD-GFP Mediates Cytotoxicity of Cry1Ac in Hi5 Cells in Contrast to SlCAD-GFP

**Figure 1:**
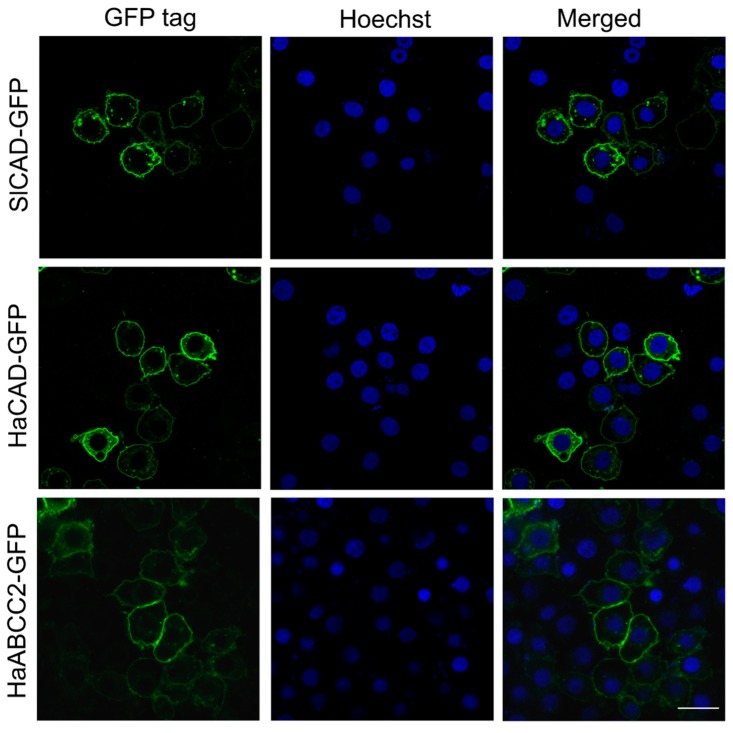
Subcellular localization of cadherin (CAD) and ABCC2 proteins with GFP tag at C terminus from different lepidopteran species expressed in Hi5 cells. The GFP fluorescence in all these CAD molecules was observed in the confocal fluorescent microscope. The nuclei were stained with Hoechst 33342 (1 µg/mL). Bar, 20 µm.

We cloned the open reading frame of *S. litura SlCAD* (GenBank: JN687590) and fused it to GFP protein as described in materials and methods. The putative amino acid sequence was deduced and aligned with the sequence of *H. armigera HaCAD* [16] using the Needleman-Wunsch algorithm of EMBOSS Needle (https://www.ebi.ac.uk/Tools/psa/emboss_needle/) showing 56% amino acid identity. Expression of HaCAD-GFP and SlCAD-GFP proteins in Hi5 cells demonstrated that both GFP tagged CAD proteins were located on the plasma membrane (Figure 1). We used CAD proteins from *S. litura* and *H. armigera* fused to GFP since these constructions were efficiently expressed in Hi5 cells (Figure 1). The cytotoxicity assays show that HaCAD-GFP was able to mediate toxicity of Cry1Ac to Hi5 cells and swollen cells were observed. In contrast, the SlCAD-GFP was not, even at the highest Cry1Ac concentration used (40 µg/mL), where healthy cells were observed, similar to the control of Hi5 cells treated with PBS buffer. The Hi5 cells expressing only the GFP protein and treated with Cry1Ac also showed healthy cells (Table 1, Figure S1).

HaCAD was also fused to FLAG-tag at N-terminus (HaCAD-Flag) (Table S1). However, this construction resulted in lower expression in the Hi5 cells than HaCAD-GFP, showing 29% of cell swelling at 40 µg/mL (Table S1). For this reason we did not work further with this construction. In contrast when the FLAG-tag was fused to ABCC2 (HaABCC2-Flag) the susceptibility to Cry1Ac was similar to the cells transfected with HaABCC2-GFP. It is important to mention that the presence of the GFP or Flag tags in CAD protein did not affect their capacity to potentiate Cry1Ac toxicity when ABCC2, was co-expressed, since both HaCAD-GFP and HaCAD-Flag induced high levels of Cry1Ac toxicity when HaABCC2 is co-transfected into the Hi5 cells (Table S1).

### 2.2. The HaCAD TB Domain is Necessary to Cooperate with HaABCC2 Resulting in High Cry1Ac Cytotoxicity

**Figure 2:**
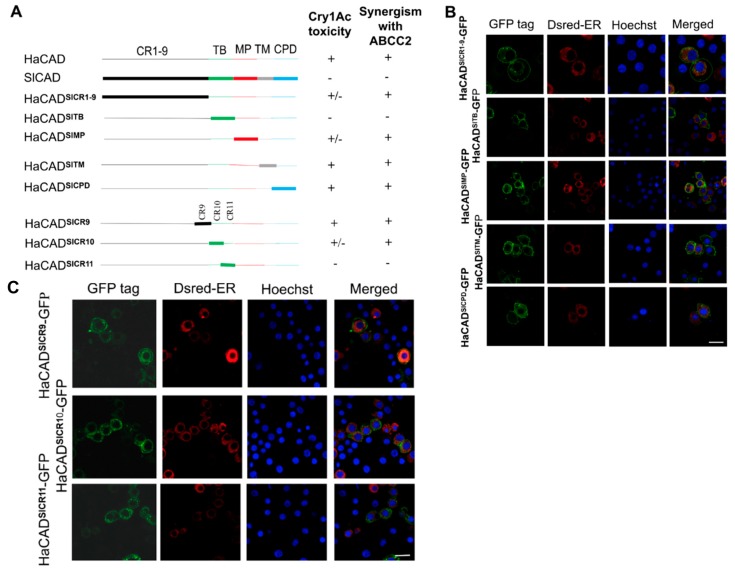
Construction of HaCAD-GFP hybrid proteins containing fragments of SlCAD and their location when expressed in Hi5 cells. **A**, show diagrams of the hybrid proteins that were constructed as well as a summary of the results for the cytotoxicity assays showing the capacity of these constructions to induce Cry1Ac toxicity in Hi5 cells and to potentiate Cry1Ac toxicity with HaABCC2-GFP (Table 2 and Table 3). **B** and **C**, show the localization of these constructions by GFP fluorescence observation. These figures show that all of them are localized in the cell surface. The endoplasmic reticulum was labeled with Dsred-ER and nuclei were stained with Hoechst 33342 (1 µg/mL). Bar, 20 µm.

**Figure 3:**
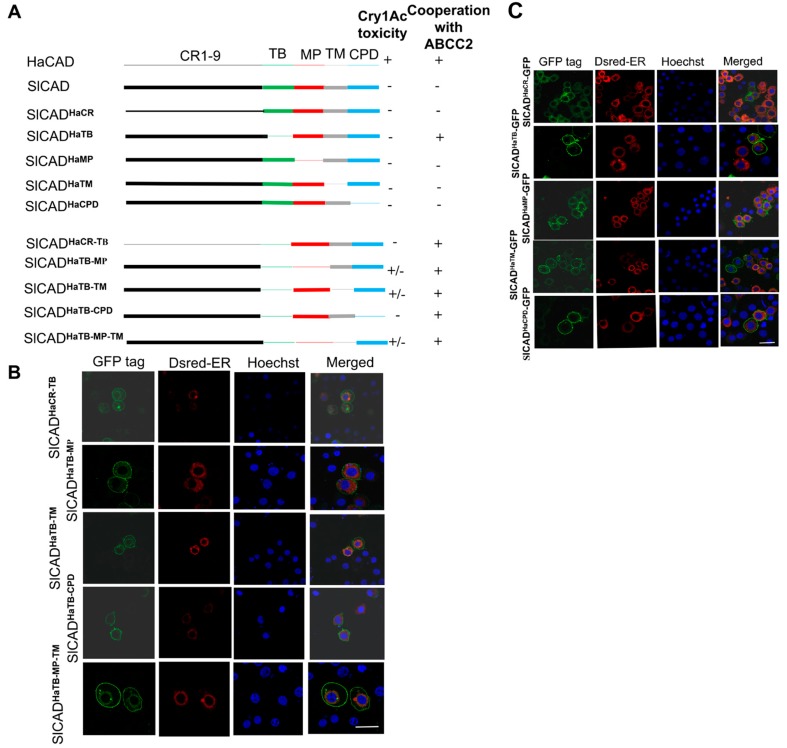
Construction of SlCAD-GFP hybrid proteins containing fragments of HaCAD and their location when expressed in Hi5 cells. **A**, showing diagrams of the hybrid proteins that were constructed as well as a summary of the results of cytotoxicity assays showing the capacity of these constructions to induce Cry1Ac toxicity in Hi5 cells and to potentiate Cry1Ac toxicity with HaABCC2-GFP (Table 2, Table 3 and Table 4). **B** and **C**, showing the localization of these constructions by GFP fluorescence observation. All of them are localized in the cell surface. The endoplasmic reticulum was labeled with Dsred-ER and nuclei were stained with Hoechst 33342 (1 µg/mL). Bar, 20 µm.

Co-expression of HaCAD-GFP and HaABCC2-GFP in Hi5 cells showed high levels of Cry1Ac toxicity, up to 735-fold higher compared to HaCAD-GFP alone (Table 1) or 28-fold higher compared to HaABCC2-GFP alone (Table 2). In contrast, the co-expression of SlCAD-GFP with HaABCC2-GFP showed no effect in the cytotoxicity of Cry1Ac (Table 2). In order to identify the regions of HaCAD-GFP that are involved in enhancing Cry1Ac toxicity with ABCC2-GFP, we substituted different regions between SlCAD-GFP and HaCAD-GFP proteins. We generated two sets of constructs (Figure 2 and Figure 3) to determine the critical regions of the HaCAD that are important to induce high levels of Cry1Ac toxicity when HaABCC2 was cotransfected in the same Hi5 cells. 

In the first set of experiments, we introduced different domains of SlCAD-GFP into the HaCAD-GFP background to determine what domain substitution results on the loss of cooperation of HaCAD with HaABCC2 for enhancing Cry1Ac toxicity. For this aim we analyzed the Cry1Ac activity in Hi5 cells transfected with these constructions in the absence and in the presence of HaABCC2. A description of all different hybrid HaCAD molecules that were constructed is shown in Figure 2A, where HaCAD-GFP domains are represented by thin lines while SlCAD-GFP domains by thick lines. These constructions were transformed into Hi5 cells and observed under confocal microscope, showing that all hybrid proteins were expressed in the cell surface of Hi5 cells (Figure 2B,C).

We analyzed if these CAD constructions were able to induce susceptibility to Cry1Ac. When the TB from HaCAD-GFP was replaced with that from SlCAD-GFP, the hybrid HaCAD-GFP^SlTB^ did not mediate cytotoxicity of Cry1Ac, even at the highest Cry1Ac concentration (40µg/mL) (Table 3). Analysis of the hybrids HaCAD-GFP^SlCR10^ and HaCAD-GFP^SlCR11^, containing the individual CR (CR10 and CR11) from SlCAD-GFP showed that both of them participated in inducing cytotoxicity of Cry1Ac (Table 3). In contrast, the substitution of HaCAD-GFP^SlCR1-9^ and HaCAD-GFP^SlMP^ showed just a small reduction in Cry1Ac susceptibility, while HaCAD-GFP^SlTM^, HaCAD-GFP^SlCPD^ and HaCAD-GFP^SlCR9^ did not affect Cry1Ac susceptibility (Table 3).

We then analyzed if these constructions were able to enhance cytotoxicity of Cry1Ac with HaABCC2-GFP. The toxicity assays showed that only the hybrid HaCAD-GFP^SlTB^ lost the capacity to enhance Cry1Ac toxicity in the presence of HaABCC2-GFP (Table 2). The other constructions containing other regions of SlCAD-GFP (HaCAD-GFP^SlCR1-9^, HaCAD-GFP^SlMP^, HaCAD-GFP^SlTM^, and HaCAD-GFP^SlCPD^) were capable to potentiate Cry1Ac toxicity with HaABCC2-GFP protein (Table 2). Analysis of the effect of the individual CR regions (CR10 and CR11) from the TB domain of SlCAD-GFP in the HaCAD-GFP background showed the CR11 from SlCAD-GFP (HaCAD-GFP^SlCR11^) was enough to prevent the potentiation effect with HaABCC2-GFP of Cry1Ac toxicity (Table 2). These results show that CR11 is the most important region of CAD protein for its cooperative interaction with ABCC2 that results in high toxicity of Cry1Ac toxin.

In the second set of experiments, we made the reverse constructions with the aim to confirm all these data. These constructions are described in Figure 3, where different domains of HaCAD-GFP were introduced into the SlCAD-GFP background. The objective was to determine which HaCAD domain was able to restore cooperation of SlCAD with HaABCC2 resulting in enhanced Cry1Ac toxicity. For this aim we also analyzed the Cry1Ac activity in Hi5 cells transfected with these constructions in the absence and in the presence of HaABCC2 (Table 3). It is important to mention that in these reverse hybrid constructions, when each domain of SlCAD-GFP was substituted with the corresponding region from HaCAD-GFP (Figure 3A), none of these hybrid SlCAD-GFP proteins with single regions from HaCAD-GFP, including the hybrid SlCAD-GFP^HaTB^ construction, was able to mediate Cry1Ac cytotoxicity even at the highest concentration of 40 µg/mL in Hi5 cells (Table 3). These results suggest that other CAD regions, besides HaTB, may be needed to induce Cry1Ac cytotoxicity.

A graphical description of all different hybrid SlCAD-GFP molecules that were constructed is shown in Figure 3A, where HaCAD-GFP domains are represented by thin lines while SlCAD-GFP domains by thick lines. These constructions were also expressed in the cell surface of Hi5 cells as shown in Figure 3B,C. The synergistic activity of SlCAD-GFP^HaTB^ with HaABCC2-GFP confirmed that the TB region of HaCAD-GFP was a key region to induce synergism and potentiate Cry1Ac toxicity, showing a 28-fold reduction in the half maximal effective concentration value of Cry1Ac toxin (EC_50_) (Table 2). The replacement of the other regions of HaCAD-GFP in the SlCAD-GFP background such as hybrids SlCAD-GFP^HaCR1-9^, SlCAD-GFP^HaMP^, SlCAD-GFP^HaTM^ and SlCAD-GFP^HaCPD^ did not induce high toxicity of Cry1Ac with HaABCC2-GFP (Table 2). Consistent with these results, hybrids of SlCAD-GFP containing additional regions from HaCAD-GFP besides the HaTB (SlCAD-GFP^HaCR1-9, TB^ or SlCAD-GFP^HaTB, MP^) were also able to enhance Cry1Ac toxicity when HaABCC2-GFP was co-transfected in Hi5 cells (Table 2). As an additional control, we constructed a hybrid SlCAD-GFP protein containing the TB region from *H. virescens* CAD (HevCAD-GFP). We selected to work with HevCAD-GFP since it was shown that this protein was also able to induce cytotoxicity of Cry1Ac when transfected into *Drosophila* S2 cells [25]. The hybrid SlCAD-GFP^HevTB^ protein was able to enhance Cry1Ac toxicity if HaABCC2-GFP was also present in the cells (Table 2). These data indicated that TB region from CAD proteins that induce Cry1Ac susceptibility, such as HaCAD-GFP and HevCAD-GFP, was determinant to show the cooperative effect with ABCC2, resulting in high potentiation of Cry1Ac toxicity.

### 2.3. Additional Regions Besides TB Region are Necessary to Induce Toxicity of Cry1Ac When ABCC2 is Absent

Since the single TB domain of HaCAD-GFP expressed in SlCAD-GFP background cannot mediate cytotoxicity of Cry1Ac on Hi5 cells, we wonder if other regions of CAD protein would be necessary to induce toxicity of Cry1Ac. Analysis of cytotoxicity of additional hybrid CAD-GFP proteins (Figure 3A) showed that the combination of TB with MP or with TM regions from HaCAD-GFP in the SlCAD-GFP background (SlCAD-GFP^HaTB, MP^, SlCAD-GFP^HaTB, TM^, SlCAD-GFP^HaTB, MP, TM^) induced cytotoxicity of Cry1Ac, resulting in more than 40% cell swelling at 40 µg of Cry1Ac per mL (Table 4).

**Figure 4:**
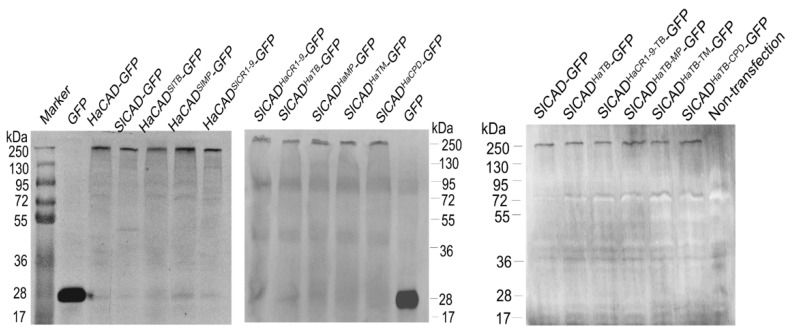
Expression levels of recombinant GFP tagged CAD proteins in Hi5 cells. The CAD-GFP fusions of wild type and hybrid CAD proteins were detected by western blot using anti-GFP polyclonal antibody and fluorescence labeled secondary antibody. All constructions showed similar expression in Hi5 cells.

Since expression levels of Bt receptors could influence the cytotoxicity of Cry1Ac, we compared the expression levels of hybrid CAD-GFP proteins with those of the wild type CAD-GFP by western blot. Figure 4 show that all hybrid CAD-GFP proteins have similar expression levels when compared with HaCAD-GFP and SlCAD-GFP. Therefore, differential susceptibility to Cry1Ac did not correlate with differences in their expression levels of these proteins.

### 2.4. The TB Fragment from HaCAD-GFP Binds Cry1Ac and TB Fragment Inhibits Cry1Ac Cytotoxicity in Hi5 Cells Transfected with HaCAD-GFP

**Figure 5:**
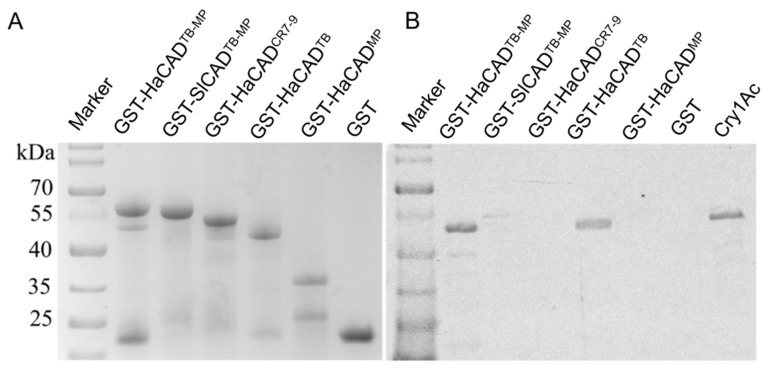
Interaction of TB and MP fragments with Cry1Ac. **A**, SDS-PAGE analysis of the purified GST tagged fragments expressed in bacteria. The protein bands were stained by Coomassie brilliant blue. **B**, Pull-down assays showing the binding of Cry1Ac to GST tagged TB-MP and to TB fragments from HaCAD but not to MP and CR7-9 fragments from HaCAD, nor to the TB-MP fragment from SlCAD. The proteins were loaded in SDS-PAGE and detected by western blot using anti-Cry1Ac antibody (1:2000).

To determine if both TB and MP regions are involved in Cry1Ac binding, different GST tagged fragments from HaCAD were used in pull-down assays of Cry1Ac toxin. The different HaCAD fragments fused to GST were expressed in *Escherichia coli* and purified. We constructed GST-TB, GST-MP, GST-TB-MP fragments from HaCAD as well as the GST-HaCAD^CR7-9^ and GST-SlCAD^TB-MP^ as control fragments (Figure 5A). These protein fragments were used in pull-down assays after incubation with Cry1Ac toxin. The data showed that the GST tagged TB fragment from HaCAD was able to bind Cry1Ac toxin. Also, as expected, GST-HaCAD^TB-MP^ from HaCAD bound Cry1Ac (Figure 5A). In contrast, the protein fragments GST-HaCAD^MP^, GST-HaCAD^CR7-9^ and the controls (GST-SlCAD^TB-MP^ and GST) did not bind to Cry1Ac (Figure 5B). These data indicate that MP domain and CR7-9 region from HaCAD are not involved in binding to Cry1Ac, only TB region bind Cry1Ac.

Finally, we analyzed the effect of these CAD fragments in the toxicity of Cry1Ac to Hi5 cells expressing HaCAD-GFP, HaABCC2-GFP or both receptors. We selected to use 1:50 mole ratio toxin: CAD fragment to assure that the soluble CAD fragments were able to compete binding of Cry1Ac with the HaCAD-GFP expressed in the Hi5 cells. The transfected cells were treated with Cry1Ac mixed with the purified CAD fragments and toxicity inhibition was determined by absence of cell swelling after treatment using the highest concentration of Cry1Ac (40 μg/mL) for 1 h. Our data indicated that the GST-TB^HaCAD^ inhibited Cry1Ac cytotoxicity in Hi5 cells expressing HaCAD-GFP and also, as expected, the GST-TB-MP^HaCAD^ inhibited Cry1Ac cytotoxicity in Hi5 cells expressing HaCAD-GFP (Table 5). The MP of HaCAD and TB-MP fragment from SlCAD did not inhibit toxicity of Cry1Ac, (Table 5). Interestingly, neither the GST-TB^HaCAD^, nor the GST-TB-MP^HaCAD^ fragment, inhibited toxicity of Cry1Ac in Hi5 cells transfected with HaABCC2-GFP or with the combination of HaCAD-GFP and HaABCC2-GFP (Table 6). These results suggest that ABCC2 interacts with an additional region of Cry1Ac that is not recognized by the TB region of CAD.

### 2.5. The CPD Region of HaCAD-GFP is not Necessary to Mediate Cry1Ac Toxicity neither to Synergize the Toxicity of Cry1Ac with ABCC2

We further constructed different HaCAD deletion mutants of each CAD domain to analyze their role in synergism and Cry1Ac toxicity (Figure S2A). However, all deletion constructs, with the exception of deletion of ΔCPD, reduced their expression in the cell surface and were accumulated in the cytoplasm (Figure S2B). In accordance with their lack of localization in the plasma membrane, these proteins were affected in their capacity to induce Cry1Ac susceptibility in Hi5 cells (Figure S2C). Deletions ΔCR_4–6_ or ΔCR_7–9_ significantly decreased cytotoxicity of Cry1Ac, while the deletion of ΔTB or ΔMP regions resulted in no-toxicity of Cry1Ac on Hi5 cells (Figure S2C). Only the deletion of CPD (HaCAD-GFP^Δ^^CPD^) was still able to induce susceptibility to Cry1Ac comparable to that of the full-length HaCAD-GFP (Figure S2C and Table S2).

Co-expression of HaCAD-GFP^ΔCPD^ with HaABCC2-GFP in Hi5 cells showed similar levels of synergism compared with the HaCAD-GFP (Table S3). The results of this experimental system confirmed that the CPD domain might not be involved in Cry1Ac toxicity and also that this region is not required to synergize the toxicity of Cry1Ac with ABCC2.

## 3. Discussion

Cry1Ac toxin relies on the binding to different larval midgut proteins for oligomerization and for its insertion into the membrane to form lytic pores. As stated above, it has been suggested that CAD binding facilitates the oligomerization of Cry1A toxins while ALP and APN binding facilitates the insertion of the toxin oligomers into the membrane. In contrast, ABCC2 participates in both oligomer formation and insertion of Cry1A oligomers into the membrane [7,9,10,11,26]. Interestingly, it has been shown that CAD and ABCC2 potentiate the toxicity of Cry1 toxins when both receptors are co-expressed in different insect cell lines [11,12,13,14,15,27].

Here we analyzed the role of the different CAD structural domains to potentiate Cry1Ac toxicity with ABCC2. We were able to identify that the HaCAD TB domain, specifically CR11 region, plays a key role in the cooperation effect with HaABCC2 resulting in 28 fold enhancement of the Cry1Ac cytotoxicity, when compared with Hi5 cell expressing only HaABCC2 (Table 2). The soluble TB fragment (containing CR10-CR11) of HaCAD expressed in *E. coli* was able to bind to Cry1Ac as shown in the pull down assays (Figure 5). However, in the absence of HaABCC2, the HaCAD TB domain expressed in the SlCAD background was not sufficient to confer susceptibility of the Hi5 cells to Cry1Ac toxin since in addition to TB other regions such as the MP or TM regions, are also needed in the SlCAD background. Interestingly Hi5 cells expressing HaCAD hybrids with SlCAD domains MP or TM, which were predicted to affect the toxicity of Cry1Ac toxin, showed that when MP from SlCAD was introduced into HaCAD background (HaCAD-GFP^SlMP^) the toxicity of Cry1Ac was substantially reduced since only 50% cell swelling was observed at the highest Cry1Ac concentration used (40 µg/mL) (Table 3). In contrast, when TM from SlCAD was introduced into HaCAD background (HaCAD-GFP^SlTM^) the toxicity of Cry1Ac was only reduced two fold (EC_50_ 13.12 µg/mL (11.62–14.87)) compared to HaCAD (EC_50_ 7.36 µg/mL (6.23–8.59)) (Table 3). These results suggest that MP region along with TB are the most important regions to induce toxicity of Cry1Ac. However, our pull-down assays demonstrated that MP domain does not bind Cry1Ac. The role of HaCAD MP and/or TM regions in mediating Cry1Ac toxicity in Hi5 cells still remains to be determined.

In the case of *M. sexta* CAD (MsCAD), it was shown that CR12 region, that corresponds to HaCAD CR11, binds Cry1Ab and was able to enhance Cry1Ab toxicity in different insect larvae. This enhancement of Cry1Ab toxicity directly correlated with an enhanced oligomerization of the toxin [9,28]. A working hypothesis to explain the CAD-ABCC2 cooperative effect to potentiate Cry1Ac toxicity in Hi5 cells is that in the presence of CAD protein that is able to bind Cry1Ac toxin, or in the presence of its TB domain, the oligomerization of Cry1Ac is enhanced. The ABCC2 would bind those oligomers and facilitates their insertion into the membrane enhancing Cry1Ac toxicity. Future experiments will reveal if enhanced Cry1Ac toxicity correlates with enhanced oligomerization.

Here, we also show that the soluble TB of HaCAD that was expressed in *E. coli* (GST-TB^HaCAD^) reduced cytotoxicity of Cry1Ac to Hi5 cell line expressing HaCAD-GFP, possibly due to a direct competition of the binding of the toxin with CAD receptor expressed in the cells, resulting in inhibition of Cry1Ac toxicity. However, the soluble TB of HaCAD (GST-TB^HaCAD^) was not able to inhibit toxicity of Cry1Ac in cells expressing HaABCC2-GFP or in cells cotransfected with HaCAD-GFP plus HaABCC2-GFP (Figure 5, Table 5 and Table 6). These data may indicate that ABCC2 recognizes an additional region of Cry1Ac that is not recognized by the CAD TB. It is known that binding of Cry1A toxins to CAD receptor is through domain II loop regions, In the case of *Spodoptera exigua* ABCC2 it was shown that domain III is also involved in Cry1A binding [29].

Previously it was reported that TB-MP region of CAD enhanced Cry1Ac toxicity when fed to the susceptible larvae. For example, the CR12-MP region of MsCAD enhanced toxicity of Cry1Ab against *M. sexta* larvae [23,30]. The TB region of *Spodoptera frugiperda* CAD (SfCAD) containing part of the MP region also enhanced toxicity of Cry1Fa in *S. frugiperda* larvae [31]. Similarly, a longer fragment form HaCAD containing CR9-CR10-CR11-MP-TM and CPD enhanced Cry1Ac toxicity in *H. armigera* [32]. It was proposed that the protection of CAD fragment from protease degradation in the midgut due to their membrane binding and enhanced pore forming activity of Cry toxin could explain the mechanism of synergism of these CAD fragments enhancing Cry1Ac toxicity [31]. However, it is important to mention that in all these examples the ABCC2 protein is present in the midgut cells of the susceptible larvae and thus a cooperative effect between the CAD TB domain with the ABCC2 protein that is present in those larvae could explain the enhanced toxicity of Cry1Ac. It will be important to determine if the CAD TB fragments enhance Cry1A toxicity in larvae lacking ABCC2. In the Hi5 cells the enhancement of Cry1Ac toxicity is only observed when co-transfected with HaABCC2-GFP and HaCAD-GFP. In agreement with this argument, no inhibition of Cry1Ac toxicity by HaCAD TB-MP or TB fragments (GST-TB-MP^HaCAD^ or GST-TB^HaCAD^) was observed when HaABCC2-GFP was expressed in Hi5 cells (Table 6). 

Finally, an alternative model of Cry1Ab mode of action was previously proposed in Hi5 cells transfected with MsCAD. This alternative model proposed that binding of Cry1Ab to MsCAD triggers an intracellular cascade signal pathway involving protein kinase A and adenylate cyclase leading to cell death [33]. This alternative model of the mode of action implies that the CPD domain of CAD is essential for the interaction with other protein components of the signal transduction pathway. In agreement with this model, it was shown that a resistant allele of *H. armigera* with a deletion in the intracellular CPD is linked with Cry1Ac resistance [34]. However, when this mutated CAD allele was expressed in Sf9 cells, the cells were able to bind Cry1Ac and also become susceptible to Cry1Ac, showing a LC_50_ value only two times higher than the wild type allele of CAD [34], suggesting that other mutations may be involved in the phenotype observed in the resistant line. Our data show that deletion of CPD domain in HaCAD-GFP^ΔCPD^ was still able to confer susceptibility of Hi5 cells to Cry1Ac and also to synergize Cry1Ac toxicity when co-expressed with HaABCC2. These data support a previous published work showing that the *Bombyx mori* CAD (BmCAD) deleted of CPD was still able to confer susceptibility to Cry1Aa and Cry1Ab toxins in Sf9 cells and was also able to potentiate Cry1Aa/b toxicity when co-expressed with BmABCC2 [24]. Overall, these data indicate that the signal transduction pathway previously reported in Hi5 cells transfected with MsCAD plays a minor role, if any, in the Cry1Ac or Cry1Aa/b mechanism of action in Hi5 cells or Sf9 cells transfected with HaCAD or BmCAD respectively. Also, that CPD region of CAD is not involved in the potentiation effect of CAD with ABCC2 to induce high toxicity of Cry1A toxins.

It has been proposed that CAD binding facilitates Cry toxin oligomerization while ABCC2 binding is involved in insertion of oligomers into the membrane [7]. Thus based on the results described here, we propose a new working hypothesis where the HaCAD TB domain recruits Cry1Ac toxin by interacting through CR11, promoting toxin oligomerization and localizing the toxin oligomers in a good position to interact with the ABCC2. In turn, the ABCC2 can induce oligomerization and insertion of the oligomer into the membrane. These events could explain the synergism between these proteins resulting in enhanced Cry1Ac toxicity, future work will follow this line.

## 4. Materials and Methods

### 4.1. Cell Lines and Cry1Ac Toxin

*Trichoplusia ni* cell line Tn-5B1-4 (Hi5) was purchased from Life Technologies Co. (Carlsbad, CA, USA) and cultured in Grace’s insect cell culture medium (Life Technologies, Carlsbad, CA, USA) supplemented with 10% fetal bovine serum (Life Technologies, Carlsbad, CA, USA), 100 U/mL penicillin (Life Technologies, Carlsbad, CA, USA) and 100 µg/mL streptomycin (Life Technologies Co.). We selected to work with *Trichoplusia ni* Hi5 cells since these cells show higher transfection efficiency than other cells lines such as Sf9 (Table S4) and showed low expression of CAD and no expression of ABCC2 transcripts from *T. ni* as determined by real time quantitative PCR (RT-qPCR) analysis (Table S5). The Hi5 cells without transfection were not susceptible to the highest concentration of Cry1Ac that was tested (40 µg/mL) showing no swollen cells.

Rabbit anti-GFP polyclonal antibody ab137827 and Rabbit anti-β-tubulin antibody were purchased from Abcam (Cambridge, UK). The rabbit anti-Cry1Ac antibody was kindly provided by Dr. Gemei Liang from Institute of Plant Protection, Chinese Academy of Agricultural Sciences, Beijing China. Dylight 800 goat anti-rabbit secondary antibody (IgG) was purchased from Abbkine Inc (Redlands, CA, USA). The purified activated and lyophilized Cry1Ac toxin was kindly donated by Dr. Marianne Pusztai-Carey from Case Western Reserve University, USA. The Cry1Ac was isolated from *B. thuringiensis* HD73 strain. The protoxin inclusion bodies were solubilized, activated by trypsin, purified by high performance anion-exchange liquid chromatography at pH 10 in an increasing gradient of sodium chloride, as previously described [35] and lyophilized. Lyophilized toxins were dissolved in 50 mM NaHCO_3_ buffer (pH 9.5) at 1 mg/mL. The pH of the solubilized toxin was adjusted to pH 7.4 using 1 M NaH_2_PO_4_ buffer (pH 7.0). Protein concentration was determined by the BCA method (Pierce, Rockford, IL). These samples were put into 200-μL Eppendorf tubes at 20 μL/tube, stored at −80 °C, and diluted in PBS (pH 7.4) before use.

### 4.2. Cloning of SlCAD

*S. litura* larvae were purchased from Keyun Co. (Jiyuan, China). Total RNA was extracted from midgut tissue of 4th instar larvae using TriZol reagent according to the manual provided by the company (Life Technologies). The cDNA was synthesized using the cDNA synthesis kit from Takara (Dalian Bio., China) and the open reading frame of *SlCAD* was amplified by PCR using specific primers (Table S4) (GenBank: JN687590). The PCR reactions were done using I-5™ 2 × High-Fidelity Master Mix (Molecular Cloning Laboratories, MCLAB, San Francisco, CA, USA) according to the following program: 98 °C for 2 min (once), followed by 30 cycles, each cycle consisting in: 53 °C for 15 s, 72 °C for 40 s and 98 °C for 10 s.

The DNA fragments encoding *H. virescens* CAD (HevCAD) TB, TM and CPD (codifying for 1216-1732 amino acid residues) (GenBank: AF367362.1) were synthesized by Genscript Co. (Nanjin, China) and inserted into pGEM-T easy vector using pEASY-Uni seamless cloning and assembly kit from Transgen Biotech (Beijing, China). The sequences were confirmed by DNA sequencing.

### 4.3. Plasmids for Protein Expression in Hi5 Insect Cells

The plasmids used for expression of GFP, HaCAD-GFP (GenBank: AF519180) and HaABCC2-GFP (GenBank: KF479231) were previously constructed in our laboratory [36,37,38]. The plasmids used for expression of SlCAD-GFP (GenBank: JN687590) and HevCAD-GFP (GenBank: AF367362) were constructed using the specific primers through the gene fusion method. It was briefly introduced as follow. The inserted fragments and the vector (pie2-HaCAD-GFP or pie2-SlCAD-GFP) fragments were amplified by PCR using the different templates, and purified using the Gel Extraction Kit (Bio-tek, Winooski, VT, USA), respectively. The purified inserted fragments were mixed with the purified vector fragments and transformed into *E. coli* DH5α. The homologous recombination occurred between the two fragments in the bacterium and the positive clones were identified by sequencing. The various deletions in *HaCAD-GFP* gene were constructed using the overlap PCR method and inserted into the plasmid pie2-EGFP-N/pGFP as previously described [36,38]. It was briefly described as follow. The up-fragment and the down-fragment of target gene were amplified by PCR using the corresponding plasmids containing the target fragments as templates, respectively. The products of PCR were run on agarose gels and the up-fragment and the down-fragments were cut from gel and purified using the Gel Extraction kit (Bio-tek, Winooski, VT, USA), respectively. The full-length gene was amplified by PCR using the mixture of the up-fragment with the down-fragment as template and the specific gene primers. The full-length PCR product was purified and digested with corresponding restriction endonuclease. Finally the digested fragments were cloned into expression plasmid pie2-EGFP-N1. The plasmids for expression of the different hybrid CAD proteins between HaCAD-GFP and SlCAD-GFP were constructed using gene-fusion through recombinase method as described above [39,40]. All primers were listed in Supplemental Tables S6–S13. For plasmid purification, plasmid DNA mini Kit was from Omega Bio-teck, Inc (Winooski, VT, USA) was used.

### 4.4. Expression and Purification of Proteins in E. coli

The plasmids for expression of the GST-TB-MP fragments from HaCAD and SlCAD and the GST-HaCAD^CR7-9^, GST-HaCAD^TB^ and GST-HaCAD^MP^ fragments from HaCAD in *E. coli* were constructed using the plasmid vector pGEX-KG and the specific primers (Table S14). Briefly, the target fragments encoding the corresponding regions (TB-MP, CR7-9, TB and MP) of cadherin protein were amplified by PCR using plasmids pie2-HaCAD-GFP or pie2-SlCAD-GFP as template and specific primers, respectively. The PCR product was run on agarose gel and the target fragments were purified using the Gel Extraction Kit (Bio-tek, Winooski, VT, USA). Then, the fragments were digested with restriction endonuclease and cloned into plasmid pGEX-KG digested by the appropriate enzymes. The positive clones were identified by sequencing. After the recombinant plasmids had been constructed, they were transformed into *E. coli* BL21. The bacteria were culture for 3 to 4 h, and the OD_600_ was about 0.5. Then IPTG was added into the culture at 0.5 mM in order to induce expression of the recombinant proteins with His or GST tag at 30℃ or 16℃ for different times. The bacteria were lysed in lysis buffer containing protease inhibitor cocktail tablets (Werk, Penzberg, Germany), and the target proteins were purified, by using glutathione-sepharose 4B resin, according to the manual supplied by the company (Pharmacia Biotech Inc., Arlington Heights, IL, USA) [41]. 

### 4.5. Transfection

Hi5 cells were seeded into six-well cell culture plates (Corning Co., Corning, NY, USA) at 4 × 10^5^ cells/well and grown over night. The next day the cells were transfected as described previously [36]. We used 2 µg of each plasmid and eight µl of FuGENE HD transfection reagent (Promega Co. Madison, WI, USA) for each well. In order to co-express CAD and HaABCC2, the plasmids expressing CAD-GFP were mixed with pHaABCC2-GFP at 1:1 (mole ratio) in Grace’s insect cell culture medium without FBS and antibiotics. At the same time, the transfection reagent FuGENE HD was also mixed with the same medium described above. Finally, the two parts were mixed together and used for co-transfection of Hi5 cells according to the manufacturer.

### 4.6. Microscopic Observation

After transfection, cells were incubated for 24 h, fixed using 4% paraformaldehyde (Sigma-Aldrich, St. Louis, MO, USA) for 15 min dissolved in 0.1 M phosphate buffered saline (PBS), and nucleus were stained with Hoechst 33342 (Sigma-Aldrich, St. Louis, MO, USA) (1 µg/mL) for 10 min, viewed under fluorescence microscope (Nikon E400, Nikon Corporation, Tokyo, Japan) or laser confocal microscope (ZEISS LSM510, Carl Zeiss Microscopy GmbH, Oberkochen, Germany), and photographed [36]. 

To calculate transfection efficiency, the green light emitted by the transfected Hi5 cells expressing GFP fusion proteins was observed after excitation with blue light and the nucleus stained with Hoechst 33342 emitted blue light were activated using UV. The cells were photographed under a fluorescence microscope and the two pictures were merged. The cells emitting green and blue lights and total cells emitting blue light were counted. The transfection efficiency was calculated by using the number of cells emitting green and blue light divided by the total number of cells.

### 4.7. Cytotoxicity Assay

The cells were seeded into 48-well cell culture plates at 5 × 10^4^ cells/well and grown overnight. Then they were transfected as described above and incubated 24 h in Grace’s medium. The cells were washed twice using PBS (135 mM NaCl, 4.7 mM KCl, 10 mM Na_2_HPO_4_, 2 mM NaH_2_PO_4_, pH 7.4). The cells were treated with activated Cry1Ac diluted in PBS at different concentrations for 1 h and photographed under an inverted fluorescent microscope (Nikon TE2000-S,Nikon Corporation, Tokyo, Japan). The cytotoxicity assays were based on analyzing cell swelling that was previously described to correlate with cell death [42]. The swollen cells became round and bigger than the normal cells (Figure S1). The swollen cells can be stained by Trypan blue indicating that they are dead cells (Figure S3). The cells were photographed after they were treated with Cry1Ac for 1 h and the percentages of the swollen cells were calculated on the pictures from at least 3 fields of the microscope.

To score the half maximal effective concentration (EC_50_) value_,_ the cells cultured in 24 well culture plates were treated with Cry1Ac using at least five different concentrations (two fold dilution) for 1 h. Then the percentage of the swollen cells was divided by the percentage of the cells emitting green fluorescence for each Cry1Ac concentration. The regression equation and concentration for 50% of maximal effect (cell swelling) (EC_50_) and 95% confidence interval (CI) were calculated using SPSS 16.0 version software (DataNet Co., Southfield, MI, USA). 

The potentiation of Cry1Ac toxicity was calculated by determining the ratio of the EC_50_ of Cry1Ac obtained in cells transfected with HaABCC2-GFP divided by the EC_50_ value obtained in cells co-expressing CAD-GFP and HaABCC2-GFP.

### 4.8. Western Blot Assay

The cells were grown into six-well cell culture plates at 4 × 10^5^ cells/well and after 36 h of transfection, they were lysed with RIPA lysis and extraction buffer as described by the manufacturer (Thermo Fisher Scientific Inc, Rockford, IL, USA). The proteins were separated on 8% SDS-PAGE gels and electrotransferred to PVDF membrane (Millipore Co., Billerica, MA, USA). The PVDF membrane was blocked with 5% non-fat milk in TBS-T (0.15 mM sodium chloride, 0.01 mM Tris-base and 0.1% tween-20, pH 8.0) for 3 h, the PVDF membrane was incubated with rabbit anti-GFP polyclonal antibody (Abcam, Cambridge, UK) diluted in TBS-T(1:1000)overnight at 4 ℃. After washing three times with TBS-T, the PVDF membrane was incubated with DyLight 800 goat anti-rabbit IgG (Abbkine, Wuhan, China) at 1:8000 dilution in TBS-T. Finally, the membrane was washed three times with TBS-T, and bands were scanned using the Odyssey system (LI-COR Bioscience, Lincoln, NE, USA).

### 4.9. Pull-down Assay

We used 800 ng of purified protein fragments per sample, GST, GST-TB-MP fragments from HaCAD (GST-HaCAD^TB-MP^), or SlCAD (GST-SlCAD^TB-MP^) and GST-MP fragment from HaCAD (GST-HaCAD^MP^), bound to glutathione matrix (Pharmacia Biotech Inc.,USA) for 3 h. After washing 3 times with PBS, the beads were incubated with the activated Cry1Ac at 40 µg/mL diluted in PBS supplemented with 600 mg/L CaCl_2_ for 3 h. The beads were washed 6 times with PBS-NaCl-Tween 20 (0.1%), and 3 times with PBS-DTT (1 mM)-Tween 20 (0.1%). The proteins bound to the beads were denatured with loading buffer and boiling for 5 min, then centrifuged at 16,000 *g* for 1 min. The proteins in this supernatant were separated on 10% SDS-PAGE gel and western blotting was carried out using rabbit anti-Cry1Ac antibody (1:2000) and goat anti-rabbit fluorescence-label secondary body (1:8000) as described above. The bands on the membrane were scanning using the Odyssey system.

### 4.10. Real Time RT-qPCR Assay

Total RNA was extracted from Hi5 cells using TRIzol reagent (Invitrogen, Carlsbad, CA, USA) after the cells had been co-transfected for co-expressing HaCAD-GFP and HaABCC2-GFP for 36 h. The first cDNA strand was synthesized using the RNA. The real time RT-PCR was performed according to the following reaction condition. The reaction mixture contained 10 μL of SYBR real-time qPCR master mix (US Everbright, Suzhou, China), 4 μL of diluted cDNA (1:50), 0.5 μL of each of the forward and reverse primers (10 μM) (Table S15), and 5 μL of PCR-grade water in a final volume of 20 μL. The following reaction conditions were applied: 3 min at 95 °C, 40 cycles of 15 s at 95 °C and 30 s at 58 °C. The relative levels of expression of *TnCAD* (Genbank accession number: JF303656), *TnABCC2* (Genbank accession number: XM026870277.1), *HaCAD-GFP* and *HaABCC2-GFP* were normalized against that of *T. ni* ribosomal protein S3A gene (*rps3A*) (Genbank accession number: XM_026884761) using the method of 2^−ΔCT^.

### 4.11. Statistical Analysis

In order to analyze a median effect concentration (EC_50_) of activated Cry1Ac to the cells, five concentrations of two-fold diluted activated Cry1Ac were used to treat the cells for 1 h, and the percentages of the swollen cells of the transfected cells were calculated as described above for each concentration (three replicates were performed in each concentration). The EC_50_ of activated Cry1Ac to the cells was obtained by Probit analysis using SPSS version 16.0.

All experiments were performed three times (3 biological repeats), and data shown in mean ± SD. A value of *p* was calculated using a Student’s *t*-test for two groups. The statistical significance of the differences among multiple groups was assessed by One-Way ANOVA using software SPSS version 16.0. *p* < 0.05 was considered as a significant difference.

## Figures and Tables

**Table 1:** Cytotoxicity of Cry1Ac mediated by cadherin (CAD) and *H. armigera* ABCC2 (HaABCC2) proteins from two lepidopteran species when expressed in Hi5 cells.

Protein	EC_50_ (μg/mL)	95% CI (μg/mL)	Slope	x^2^	df	Susceptibility
SlCAD-GFP	>40 ^a^	N	N	N	N	−
HaCAD-GFP	7.36	6.23–8.59	3.11	2.90	3	+
HaABCC2-GFP	0.26	0.15–0.44	4.09	13.27	3	+

^a^, The EC_50_ of Cry1Ac in cells transfected with SlCAD-GFP cannot be calculated since the percentage of cell swelling treated with maximum Cry1Ac concentration of 40 µg/mL for 1 h was less than 5%. N, not determined; −, no susceptible to Cry1Ac; +, susceptible to Cry1Ac. The number of analyzed cells emitting green fluorescence was about 300 to 800 in each group.

**Table 2:** The cooperation effect of different CAD and hybrid CAD proteins with HaABCC2-GFP resulting in potentiation of Cry1Ac cytotoxicity in Hi5 cells.

Protein	EC_50_ (µg/mL)	95% CI (µg/mL)	Slope	x^2^	df	Potentiation of Cry1Ac toxicity
GFP (control) ^a^	>40 ^a^	N	N	N	N	N
HaABCC2-GFP	0.26	0.15–0.44	4.09	13.27	3	N
GFP+HaABCC2-GFP	0.28	0.23–0.34	3.00	0.63	3	−
SlCAD-GFP+HaABCC2-GFP	0.28	0.24–0.35	3.03	1.16	3	−
HaCAD-GFP+HaABCC2-GFP	0.01	0.01–0.02	3.33	11.17	3	+ (28 fold)
HaCAD-GFP^SlCR1-9^+HaABCC2-GFP	0.02	0.02–0.03	3.24	1.54	3	+ (14 fold)
HaCAD-GFP^SlTB^+HaABCC2-GFP	0.28	0.24–0.33	3.47	1.81	3	−
HaCAD-GFP^SlMP^+HaABCC2-GFP	0.01	0.01–0.03	2.99	8.81	3	+ (28 fold)
HaCAD-GFP^SlTM^+HaABCC2-GFP	0.01	0.004–0.02	3.30	8.6	3	+ (28 fold)
HaCAD-GFP^SlCPD^+HaABCC2-GFP	0.02	0.01–0.02	3.11	2.39	3	+ (14 fold)
HaCAD-GFP^SlCR9^+HaABCC2-GFP	0.02	0.01–0.04	2.82	8.99	3	+ (14 fold)
HaCAD-GFP^SlCR10^+HaABCC2-GFP	0.03	0.02–0.03	2.70	3.44	3	+ (9.3 fold)
HaCAD-GFP^SlCR11^+HaABCC2-GFP	0.20	0.09–0.39	3.35	14.87	3	−
SlCAD-GFP^HaCR1-9^+HaABCC2-GFP	0.20	0.14–0.29	4.22	7.61	3	−
SlCAD-GFP^HaTB^+HaABCC2-GFP	0.01	0.01–0.01	5.11	3.31	3	+ (28 fold)
SlCAD-GFP^HaMP^+HaABCC2-GFP	0.21	0.14–0.29	4.41	7.35	3	−
SlCAD-GFP^HaTM^+HaABCC2-GFP	0.30	0.19–0.47	5.18	12.74	3	−
SlCAD-GFP^HaCPD^+HaABCC2-GFP	0.20	0.18–0.22	5.02	3.75	3	−
SlCAD-GFP^HaCR1-9,TB^+HaABCC2-GFP	0.01	0.005–0.02	3.00	6.26	3	+(28 fold)
SlCAD-GFP ^HaTB, MP^+HaABCC2-GFP	0.01	0.01–0.02	4.42	4.31	3	+ (28 fold)
SlCAD-GFP^HevTB^ + GFP ^a^	>40 ^a^	N	N	N	N	N
SlCAD-GFP^HevTB^+HaABCC2-GFP	0.02	0.02–0.03	5.77	3.62	3	+ (14 fold)

^a^, The EC_50_ value of Cry1Ac in cells transfected with GFP can not be calculated since the percentage of Hi5 cell swelling treated with Cry1Ac at 40 μg/mL for 1 h was less than 5%. N, not determined; +, effective potentiation effect in Cry1Ac toxicity; −, no potentiation effect of Cry1Ac toxicity. The number of cells emitting green fluorescence was about 300 to 800 in each group.

**Table 3:** Cytotoxicity of Cry1Ac mediated by hybrid CAD proteins in Hi5 cells.

Protein	% cell swelling at 40 μg/mL	EC_50_ (μg/mL)	95% CI (μg/mL)	Slope	x^2^	df	Susceptibility
GFP (control) ^a^	2.11 ± 1.17	N	N	N	N	N	−
SlCAD-GFP ^a^	2.13 ± 0.32	N	N	N	N	N	−
HaCAD-GFP	96.23 ± 3.66	7.36	6.23–8.59	3.11	2.90	3	+
HaCAD-GFP^SlCR1-9^	44.66 ± 1.15	N	N	N	N	N	+/−
HaCAD-GFP^SlTBa^	1.30 ± 0.53	N	N	N	N	N	−
HaCAD-GFP^SlMP^	50.00 ± 4.58	N	N	N	N	N	+/−
HaCAD-GFP^SlTM^	92.83 ± 1.41	13.12	11.62–14.87	4.48	0.249	3	+
HaCAD-GFP^SlCPD^	81.64 + 6.41	8.65	7.35–10.13	3.03	3.65	3	+
HaCAD-GFP^SlCR9^	81.92 ± 9.34	10.78	9.32–12.5	3.40	3.03	3	+
HaCAD-GFP^SlCR10^	16.07 ± 4.70	N	N	N	N	N	+/−
HaCAD-GFP^SlCR11^	6.23 ± 1.68	N	N	N	N	N	−
SlCAD-GFP^HaCRa^	1.95 ± 0.82	N	N	N	N	N	−
SlCAD-GFP^HaTBa^	4.73 ± 0.70	N	N	N	N	N	−
SlCAD-GFP^HaMPa^	2.86 ± 0.47	N	N	N	N	N	−
SlCAD-GFP^HaTMa^	4.91 ± 0.75	N	N	N	N	N	−
SlCAD-GFP^HaCPDa^	1.64 ± 0.55	N	N	N	N	N	−
SlCAD-GFP^HevTBa^	No cell swelling^a^	N	N	N	N	N	−

^a^, No cell swelling was observed; +, Cry1Ac susceptible; −, non-susceptible to Cry1Ac. The number of the cells emitting green fluorescence was about 300 to 800 in each group.

**Table 4:** Influences of the expression of TB from HaCAD with other domains of HaCAD in the hybrid CAD proteins on the cytotoxicity of Cry1Ac.

Protein	% Cell Swelling at 40 μg/mL	Susceptibility
SlCAD-GFP^HaTB a^	1.33 ± 0.57	−
SlCAD-GFP^HaTB, MP^	48.60 ± 1.79	+/−
SlCAD-GFP^HaTB, TM^	46.31 ± 3.96	+/−
SlCAD-GFP^HaTB, CPD a^	4.71 ± 3.01	−
SlCAD-GFP^HaCR-TB, TM^	42.19 ± 3.97	+/−
SlCAD-GFP^HaTB, MP, TM^	52.87 ± 1.86	+/−

^a^, The percentage of Hi5 cell swelling treated with Cry1Ac at 40 µg/mL for 1 h was less than 5%. +, susceptible to Cry1Ac; −, non-susceptible to Cry1Ac. The number of cells emitting green fluorescence was about 300 to 800 in each group. +/− indicates low susceptibility of Hi5 cells expressing different SlCAD hybrids. − indicates no susceptibility of Hi5 cells expressing different SlCAD hybrids.

**Table 5:** Cytotoxicity of the mixture of Cry1Ac with CAD fragments expressed in *E. coli* bacteria (1:50 mole ratio; toxin: protein fragment) in Hi5 cells expressing HaCAD.

Mixture	EC_50_ (µg/mL)	95% of CI (µg/mL)	Slope	x^2^	df	Inhibition
Cry1Ac+GST	12.96	7.75–23.14	4.38	14.51	3	−
Cry1Ac+GST-TB-MP^HaCAD^	>40 ^a^	N	N	N	N	+
Cry1Ac+GST-TB-MP^SlCAD^	15.38	13.21–18.12	3.25	2.53	3	−
Cry1Ac+GST-MP^HaCAD^	14.35	12.60–16.43	4.07	0.61	3	−
Cry1Ac+GST-TB^HaCAD^	>40 ^a^	N	N	N	N	+

^a^, The EC_50_ value of Cry1Ac cannot be calculated since the percentages of the Hi5 cell swelling treated with Cry1Ac at 40 μg/mL for 1 h was less than 5%. N, not determined; +, inhibition of Cry1Ac toxicity; −, no inhibition of Cry1Ac toxicity. The number of cells emitting green fluorescence was about 300 to 800 in each group.

**Table 6:** Cytoxicity of the mixture of Cry1Ac with different CAD fragments expressed in *E. coli* bacteria (1:50 mole ratio; toxin: protein fragment) in Hi5 cells expressing HaABCC2-GFP or co-expressing HaCAD-GFP and HaABCC2-GFP.

Mixture	Expressed Receptor in Hi5 cells	EC_50_ (µg/mL)	95% of CI (µg/mL)	Slope	*x* ^2^	df	Inhibition
Cry1Ac+GST	HaABCC2-GFP	0.15	0.07–0.25	3.15	11.04	3	−
Cry1Ac+GST	HaCAD-GFP+HaABCC2-GFP	0.013	0.010–0.016	2.39	3.32	3	−
Cry1Ac+GST-TB^HaCAD^	HaABCC2-GFP	0.11	0.01–0.21	2.62	15.12	3	−
Cry1Ac+GST-TB^HaCAD^	HaCAD-GFP+HaABCC2-GFP	0.02	0.01–0.03	2.30	14.61	3	−
Cry1Ac+GST-TB-MP^HaCAD^	HaABCC2-GFP	0.15	0.06–0.28	3.12	13.96	3	−
Cry1Ac+GST-TB-MP^HaCAD^	HaCAD-GFP+HaABCC2-GFP	0.014	0.005–0.028	2.37	10.51	3	−
Cry1Ac+GST-MP^HaCAD^	HaABCC2-GFP	0.19	0.06–0.44	3.54	21.68	3	−
Cry1Ac+GST-MP^HaCAD^	HaCAD-GFP+HaABCC2-GFP	0.013	0.011–0.015	3.96	3.66	3	−

−, no inhibition of Cry1Ac toxicity. The number of cells emitting green fluorescence was about 300 to 800 in each group.

## Supplementary Materials

The following are available online at https://www.mdpi.com/2072-6651/11/9/538/s1, Figure S1: Images of Hi5 cells expressing GFP or GFP tagged cadherin with Cry1Ac treatment, Figure S2: Construction of CAD proteins deleted of some regions, Figure S3: Staining with Trypan blue showing that the swollen cells were dead, Table S1: Cytotoxicity of Cry1Ac mediated by Cry1Ac receptors with different tags, Table S2: Influences of the deletion of domains of HaCAD on cytotoxicity of Cry1Ac on Hi5 cells, Table S3: The potentiation effect on Cry1Ac toxicity when the deleted HaCAD-GFP proteins were co-expressed with HaABCC2-GFP, Table S4: Transfection efficiency of Hi5 and Sf9 cells, Table S5: Analysis of the CAD and ABCC2 expression in Hi5 cells by RT-qPCR, Table S6: Sequence of primers used for cloning CAD proteins genes from different lepidopteran, Table S7: Sequence of primers used for PCR amplification of the deleted HaCAD fragments, Table S8: Sequence of primers used for amplification of short inserted fragments of SlCAD, Table S9: Sequence of primers used for amplification of long vector fragments of HaCAD, Table S10: Sequence of primers used primers for amplification of short inserted fragments of HaCAD, Table S11. Sequence of primers used for amplification of long vector fragments of SlCAD, Table S12: Sequence of primers used for amplification of short inserted fragments of HevCAD, Table S13: Sequence of primers used for amplification of long vector fragments of SlCAD, Table S14: Sequence of primers used for expression of GST tagged CAD fragments in *E.coli* bacteria, Table S15: Sequences of the primers used for real time qPCR analyzes.
