# Supplementary material for: The Cadherin Cry1Ac Binding-Region is Necessary for the Cooperative Effect with ABCC2 Transporter Enhancing Insecticidal Activity of Bacillus thuringiensis Cry1Ac Toxin
Source: Toxins (Basel). 2019 Sep 14;11(9):538. doi: 10.3390/toxins11090538 (PMC6784258; doi:10.3390/toxins11090538)
Supplement: Supplementary file 1 [file toxins-11-00538-s001.pdf]

Supplementary data: The cadherin Cry1Ac binding-region is necessary for the cooperative effect with ABCC2 transporter enhancing insecticidal activity of Cry1Ac toxin from *Bacillus thuringiensis*

Yuemin Ma, Jianfeng Zhang, Yutao Xiao, Yanchao Yang, Chenxi Liu, Rong Peng, Yongbo Yang, Alejandra Bravo, Mario Soberón and Kaiyu Liu

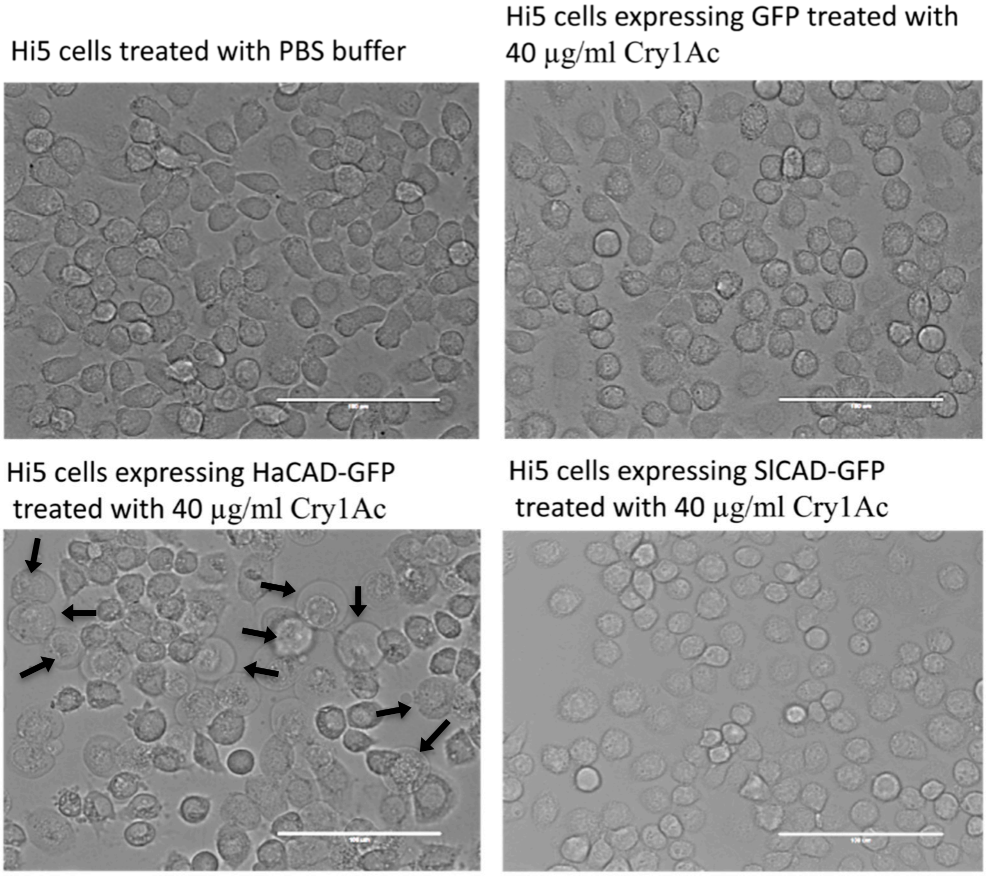

Figure S1. Images of Hi5 cells expressing GFP or GFP tagged CAD with Cry1Ac treatment. The Hi5 cells were treated with or without Cry1Ac at the indicated concentration for 1 h after they had been transfected for 24 h. Only Hi5 cells expressing HaCAD-GFP treated with a 40 μg/mL concentration of Cry1Ac showed cell swelling (arrows). Bar, 100 μm.

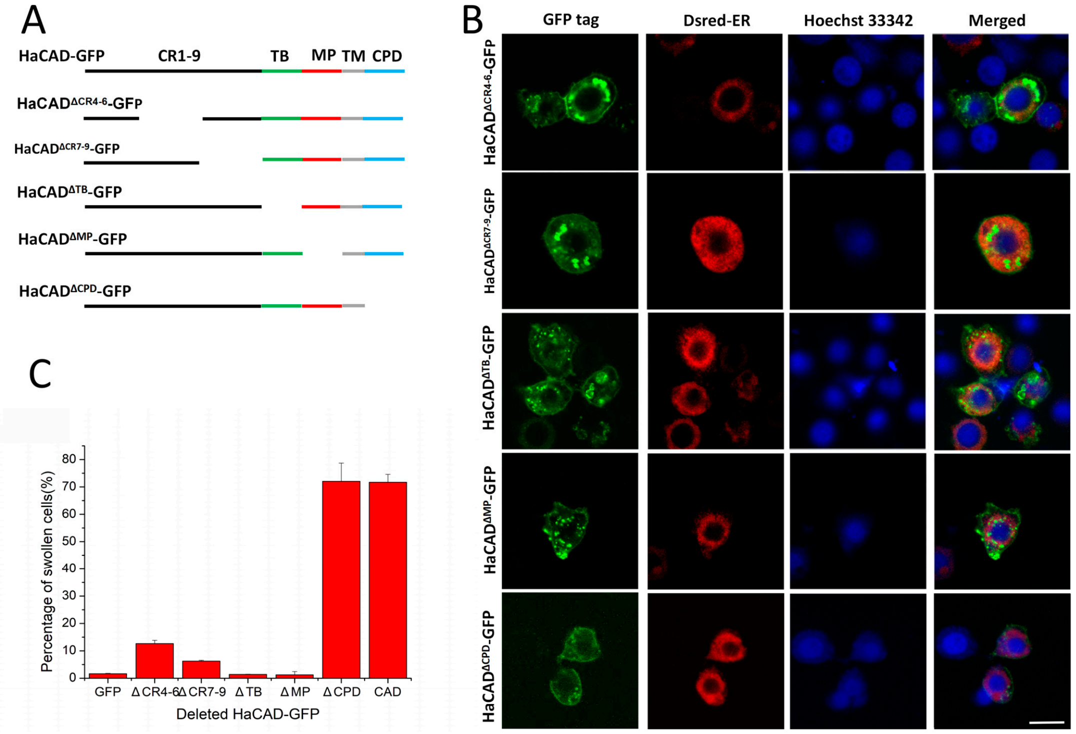

Figure S2. Construction of CAD proteins deleted of some regions**. A**, shows a diagram explaining the different deleted proteins that were constructed. **B**, shows the localization of these constructions observing GFP fluorescence. Only HaCAD-GFP$^{\Delta CPD}$ localized in the membrane. The rest of the deleted CAD proteins were localized mainly in cytoplasm. The endoplasmic reticulum was labeled with Dsred-ER and nuclei were stained with Hoechst 33342 (1 μg/mL). **C**, shows percentage of swollen cells after treatment with the different deletion CAD constructions with 40 μg/mL Cry1Ac for 1 h. The number of the cells emitting green fluorescence was about 300 to 800 in each group. Bar, 20 μm.

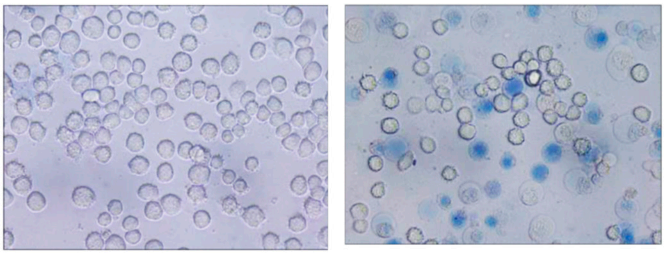

Figure S3. Staining with Trypan blue showing that the swollen cells were dead. Hi5 cells expressing HaCAD-GFP were cultured into 24-well plates and then treated with PBS buffer or Cry1Ac at 40 μg/mL for 1 h, the solution containing toxin was removed and the Trypan blue solution (0.4%, *w*/*v*) was added into wells and incubated for 5 min. The cells were observed and photographed under optical microscope.

Table S1. Cytotoxicity of Cry1Ac mediated by Cry1Ac receptors with different tags.

	Percentage of swollen cells at 40 μg/mL （%）	Slope	EC_{50} (μg/mL)	95% CI	x^2	df
HaCAD-GFP	N [a]	1.484(1.189–1.778)	7.223	5.887–8.686	1.766	3
HaABCC2-GFP	N	1.749(1.442–2.055)	0.127	0.108–0.149	3.996	3
HaCAD-GFP+HaABCC2-GFP	N	2.219(1.860–2.578)	0.010	0.006–0.014	7.126	3
HaCAD-Flag	29.02 ± 10.7	N	N	N	N	N
HaABCC2-Flag	N	1.698(1.395–2.001)	0.267	0.234–0.326	1.906	3
HaCAD-Flag+HaABCC2-Flag	N	2.439(2.076–2.802)	0.013	0.012–0.015	1.971	3

[a] N, not tested. When the GFP tag was replaced with Flag tag, the expression levels of the fusion HaCAD-Flag decreased. The EC_{50} of Cry1Ac in cells transfected with HaCAD-Flag can not be calculated since the percentage of cell swelling treated with maximum Cry1Ac concentration of 40 μg/mL for 1 h was 29%.

Table S2. Influences of the deletion of domains of HaCAD on cytotoxicity of Cry1Ac on Hi5 cells.

Protein	EC_{50}(μg/mL)	95% of CI (μg/mL)	Slope	x^2	df
GFP	>40 [a]	N	N	N	N
HaCAD-GFP	7.35	6.23–8.59	3.11	2.90	3
HaCAD$^{\Delta CPD}$-GFP	8.35	7.11–9.75	3.11	3.16	3

[a] The EC_{50} of Cry1Ac in cells transfected with GFP can not be calculated since the percentage of the aberrant Hi5 cells treated with Cry1Ac at 40 μg/mL for 1 h were less than 5%. N, not determined. The number of the cells emitting green fluorescence was about 300 to 800 in each group.

Table S3. The potentiation effect on Cry1Ac toxicity when the deleted HaCAD-GFP proteins were co-expressed with HaABCC2-GFP.

Protein	EC_{50} (μg/mL)	95% CI (μg/mL)	Slope	x^2	df	Synergism
GFP	>40 [a]	N	N	N	N	N
GFP+HaABCC2-GFP	0.28	0.23–0.34	3.00	0.63	3	—
HaCAD-GFP+ HaABCC2-GFP	0.01	0.01–0.02	3.33	11.17	3	+
HaCAD$^{\Delta CR4\text{-}6}$-GFP+HaABCC2-GFP	0.06	0.05–0.06	3.95	1.80	3	+
HaCAD$^{\Delta CR7\text{-}9}$-GFP+HaABCC2-GFP	0.06	0.04–0.09	3.35	6.00	3	+
HaCAD$^{\Delta TB}$-GFP+HaABCC2-GFP	0.27	0.21–0.36	5.25	6.02	3	—

HaCAD$^{\Delta MP}$-GFP+HaABCC2-GFP	0.22	0.15–0.32	3.96	7.04	3	—
HaCAD$^{\Delta CPD}$-GFP+HaABCC2-GFP	0.02	0.01–0.05	2.85	12.81	3	+

[a] The EC_{50} of Cry1Ac in cells transfected with GFP can not be calculated since the percentage of the aberrant Hi5 cells treated with Cry1Ac at 40 μg/mL for 1 h was less than 5%. N, not determined; –, no synergism; +, synergism.The number of the cells emitting green fluorescence was 300 to 800 in each group.

Table S4. Transfection efficiency of Hi5 and Sf9 cells.

plasmid	Hi5 (M ± SD)%	Sf9 (M ± SD)%
pHaCAD-GFP	63.1 ± 3.4 [a]	33.7 ± 5.1 [b]
pSlCAD-GFP	60.8 ± 1.9 [a]	32.6 ± 5.0 [b]
pHaABCC2-GFP	54.5 ± 7.9 [a]	31.6 ± 2.0 [b]

N = 250–280 cells each group. The positive transfected cells emitted green fluorescence under fluorescence microscope. The different upper-letters showed significant differences in the same row. M ± SD, mean ± standard deviation.

Table S5. Analysis of the CAD and ABCC2 expression in Hi5 cells by RT-qPCR.

Gene	Expression level （M±SD）
TnCAD	$(6.6 \pm 1.2) \times 10^{-5}$ [a]
TnABCC2	0 [b]
HaCAD-GFP	5.4 ± 2.1 [c]
HaABCC2-GFP	3.4 ± 0.7 [c]

HaCAD-GFP and HaABCC2-GFP were co-expressed in Hi5 cells through co-transfection using the two plasmids. The expression levels of four genes were quantified by real time RT- qPCR using the method of $2^{-\Delta CT}$ at 36 h post co-transfection. *T. ni rps3A* gene was used as an internal gene and its expression level was set as 1. The primers were listed in table S15. The different upper-letters showed significant differences in the same row. M ± SD, mean ± standard deviation.

Table S6. Sequence of primers used for cloning CAD proteins genes from different lepidopteran.

Primer	Sequence (5′-3′)	Expressed protein
SlCAD-F	AGATCTCGAGCTCAAGCTTCGGCCACCATGGCGCTTGATGTGCGAT	SlCAD-GFP
SlCAD-R	GGTGGCGACCGGTGGATCACCTCCGCCACCGCCTCCACTTTTAAACTGGGAGTTCAC	
HaCAD-F	AGATCTCGAGCTCAAGCTTCGGCCACCATGGCAGTCGACGTGAGAA	HaCAD-GFP
HaCAD-R	GGTGGCGACCGGTGGATCACCTCCGCCACCGCCTCCTCTTCTGAACTGCGTGTTCGC	
SeCAD-F	AGATCTCGAGCTCAAGCTTCGGCCACCATGGCGGTCGACGTGCGAATACT	SeCAD-GFP
SeCAD-R	GGTGGCGACCGGTGGATCACCTCCGCCACCGCCTCCTCTTCTAAACTGGGAGTTCGCG	

Table S7. Sequence of primers used for PCR amplification of the deleted *HaCAD* fragments.

Primer	Sequence (5′-3′)	Expressed protein
HaCAD-up-F	CCCTCGAGGCCACCATGGCAGTCGACGTGAGAA	Used for overlaping PCR
HaCAD-down-R	TCCCCGCGGACCTCCGCCACCGCCTCCTCTTCTGAACTGCGT GTTCGC	Used for overlaping PCR
$HaCAD^{\Delta CR4\text{-}6}$-down-F	GGGATGTTGGTGATAAAGTCGTCACTGAGTGTCCTCC	$HaCAD^{\Delta CR4\text{-}6}$GFP
$HaCAD^{\Delta CR4\text{-}6}$-up-R	GACTTTATCACCAACATCCCGGTCCTTCGCCAC	
$HaCAD^{\Delta CR7\text{-}9}$-down-F	GTGTGACCTCCTCACTACGATTCGTGCTACGG	$HaCAD^{\Delta CR7\text{-}9}$-GFP
$HaCAD^{\Delta CR7\text{-}9}$-up-R	AGTGAGGAGGTCACACTTGTCACTAGCTATG	
$HaCAD^{\Delta TB}$-down-F	CGAGAGTTCCAGGTGATATCGGACCGCAACCG	$HaCAD^{\Delta TB}$-GFP

HaCAD$^{\Delta TB}$-up-R	CACCTGGAACTCTCGGAAGTCTTCAGGGAAG	
HaCAD$^{\Delta MP}$-down-F	GAAGTGAAGGTGTACCGAGCTCTGTACGCGCTGG	HaCAD$^{\Delta MP}$-GFP
HaCAD$^{\Delta MP}$-up-R	GTACACCTTCACTTCCGTGCGGGCAGTTTC	
HaCAD$^{\Delta CPD}$-down-F	CCCTCGAGGCCACCATGGCAGTCGACGTGAGAA	HaCAD$^{\Delta CPD}$-GFP
HaCAD$^{\Delta CPD}$-up-R	GGACCGCGGTGCCCCTCCACCGCCAGTCCTAACAAAGAAC ACAATG	

Note: The two fragments (up-fragment and down-fragment) were amplified. Then the overlaping PCR was carried out using the mixture of up-fragment and down-fragment as described previously [1].

Table S8. Sequence of primers used for amplification of short inserted fragments of *SlCAD.*

Primer	Sequence (5′-3′)	Expressed protein
HaCAD^{SlCR}-F	ACAGTTTGAGTTGACGATTCGTGCTACGGACG	HaCAD^{SlCR}-GFP
HaCAD^{SlCR}-R	CCGCCACCGCCTCCTCTTCTGAACTGCGTGTTCGC	
HaCAD^{SlTB}-F	AGAGTTCCAGGTGGTTATTCGTGCCACGGATG	HaCAD^{SlTB}-GFP
HaCAD^{SlTB}-R	TGCGGTCCGATATCATGTAGATTATAACTTTT	
HaCAD^{SlMP} -F	GAAGGTGTACCTGATATCGACACGTGTCAGAG	HaCAD^{SlMP}-GFP
HaCAD^{SlMP}-R	CCGCCAGCGCGTAAACCGCCAGGGCGTAATCT	
HaCAD^{SlTM}-F	GGCGCGAGCTCTGTATATCCTCGCCGGTATCG	HaCAD^{SlTM}-GFP
HaCAD^{SlTM}-R	TTAGTGTCCTAGTCCTGATGAAGAAAGCGATG	
HaCAD^{SlCPD}-F	GTTCTTTGTTAGGAACCGAACGTTGAATCGCC	HaCAD^{SlCPD}-GFP
HaCAD^{SlCPD}-R	CCGCCACCGCCTCCACTTTTAAACTGGGAGTTCAC	
HaCAD^{SlCR9}-F	ACTGGCTACGGTAGACGGAGAGTTCCTCGACCGACTCTCCGCTA	HaCAD^{SlCR9}-GFP
HaCAD^{SlCR9}-R	ACATGATGTTCCTCGGGTCCTCGGCTTGAGGCAGCTCGAACCTC	
HaCAD^{SlCR10}-F	GGAACTACCTCGCGCCTCAGACCCCAAGAACTACAGGTGTACGG	HaCAD^{SlCR10}-GFP
HaCAD^{SlCR10}-R	GATTTCTTTCGATGAAATCTGTGTGTAAGATGCCAGCAGTGTAA	
HaCAD^{SlCR11}-F	AGCTGGCATCTCTGCTGGCGATAACGTACACAGGGATCTCATTT	HaCAD^{SlCBR11}-GFP
HaCAD^{SlCR11}-R	CTCGGTTGCGGTCCGATATCATGTAGATTATAACTTTTGCTCGA	

Note: The short inserted fragment and the following long vector fragment were used for gene fusion [2].

Table S9. Sequence of primers used for amplification of long vector fragments of *HaCAD.*

Primer	Sequence (5′-3′)	Expressed protein
HaCAD^{SlCR}-F	GCGAACACGCAGTTCAGAAGAGGAGGCGGTGGCGG	HaCAD^{SlCR}-GFP
HaCAD^{SlCR}-R	TAGCACGAATCGTCAACTCAAACTGTTGGACA	
HaCAD^{SlTB}-F	TATAATCTACATGATATCGGACCGCAACCGAG	HaCAD^{SlTB}-GFP
HaCAD^{SlTB}-R	TGGCACGAATAACCACCTGGAACTCTCGGAAG	
HaCAD^{SlMP}-F	GTGTAACATAGACCAGACGTGGTGGGCCAGCG	HaCAD^{SlMP}-GFP
HaCAD^{SlMP}-R	CACGTGTCGATATCAGGTACACCTTCACTTCC	
HaCAD^{SlTM}-F	TTTCTTCATCAGGACTAGGACACTAAACCGTC	HaCAD^{SlTM}-GFP
HaCAD^{SlTM}-R	CGGCGAGGATATACAGAGCTCGCGCCTGCGCG	
HaCAD^{SlCPD}-F	GTGAACTCCCAGTTTAAAAGTGGAGGCGGTGGCGG	HaCAD^{SlCPD}-GFP
HaCAD^{SlCPD}-R	TCAACGTTCGGTTCCTAACAAAGAACACAATG	
HaCAD^{SlCR9}-F	CGAGCTGCCTCAAGCCGAGGACCCGAGGAACATCATGTGTGAAG	HaCAD^{SlCR9}-GFP
HaCAD^{SlCR9}-R	AGAGTCGGTCGAGGAACTCTCCGTCTACCGTAGCCAGTATACCG	
HaCAD^{SlCR10}-F	TGCTGGCATCTTACACACAGATTTCATCGAAAGAAATCTGCTGA	HaCAD^{SlCBD10}-GFP
HaCAD^{SlCR10}-R	ACCTGTAGTTCTTGGGGTCTGAGGCGCGAGGTAGTTCCGCCCTC	
HaCAD^{SlCR11}-F	AAAAGTTATAATCTACATGATATCGGACCGCAACCGAGTGTTCT	HaCAD^{SlCBD11}-GFP
HaCAD^{SlCR11}-R	GATCCCTGTGTACGTTATCGCCAGCAGAGATGCCAGCTGTGTAG	

Note: The short inserted fragment and the following long vector fragment were used for gene fusion [2].

Table S10. Sequence of primers used primers for amplification of short inserted fragments of *HaCAD*.

**Primer**	**Sequence (5′-3′)**	**Expressed protein**
SlCAD^{HaCR}-F	AGAGTTCCAGGTGGTTATTCGTGCCACGGATG	SlCAD^{HaCR}-GFP
SlCAD^{HaCR}-R	CCGCCACCGCCTCCACTTTTAAACTGGGAGTTCAC	
SlCAD^{HaTB}-F	ACAGTTTGAGTTGACGATTCGTGCTACGGACG	SlCAD^{HaTB}-GFP
SlCAD^{HaTB}-R	CACGTGTCGATATCAGGTACACCTTCACTTCC	
SlCAD^{HaMP}-F	TATAATCTACATGATATCGGACCGCAACCGAG	SlCAD^{HaMP}-GFP
SlCAD^{HaMP}-R	CGGCGAGGATATACAGAGCTCGCGCCTGCGCG	
SlCAD^{HaTM}-F	AGATTACGCCCTGGCGGTTTACGCGCTGGCGGCGGTAGCTGCGG	SlCAD^{HaTM}-GFP
SlCAD^{HaTM}-R	GGCGATTCAACGTTCGGTTCCTAACAAAGAACACAATGAGCAGC	
SlCAD^{HaCPD}-F	CATCGCTTTCTTCATCAGGACTAGGACACTAAACCGTCGCTTGC	SlCAD^{HaCPD}-GFP
SlCAD^{HaCPD}-R	CCGCCACCGCCTCCTCTTCTGAACTGCGTGTTCGC	
SlCAD$^{HaCR-TB}$-F	GAAGGTGTACCTGATATCGACACGTGTCAGAG	SlCAD$^{HaCR-TB}$-GFP
SlCAD$^{HaCR-TB}$-R	CCGCCACCGCCTCCACTTTTAAACTGGGAGTTCAC	
SlCAD$^{HaTB-MP}$-F	ACAGTTTGAGTTGACGATTCGTGCTACGGACG	SlCAD$^{HaTB-MP}$-GFP
SlCAD$^{HaTB-MP}$-R	CGGCGAGGATATACAGAGCTCGCGCCTGCGCG	
SlCAD$^{HaTB-TM}$-F(SlCAD^{HaTM} was used as a background）	ACAGTTTGAGTTGACGATTCGTGCTACGGACG	SlCAD$^{HaTB-TM}$-GFP
SlCAD$^{HaTB-TM}$-R	CACGTGTCGATATCAGGTACACCTTCACTTCC	
SlCAD$^{HaTB-CPD}$-F(SlCAD^{HaCPD} was used as a background）	ACAGTTTGAGTTGACGATTCGTGCTACGGACG	SlCAD$^{HaTB-CPD}$-GFP
SlCAD$^{HaTB-CPD}$-R	CACGTGTCGATATCAGGTACACCTTCACTTCC	

Table S11. Sequence of primers used for amplification of long vector fragments of *SlCAD*.

Primer	Sequence (5′-3′)	Expressed protein
SlCAD^{HaCR}-F	GTGAACTCCCAGTTTAAAAGTGGAGGCGGTGGCGG	SlCAD^{HaCR}-GFP
SlCAD^{HaCR}-R	TGGCACGAATAACCACCTGGAACTCTCGGAAG	
SlCAD^{HaTB}-F	GAAGGTGTACCTGATATCGACACGTGTCAGAG	SlCAD^{HaTB}-GFP
SlCAD^{HaTB}-R	TAGCACGAATCGTCAACTCAAACTGTTGGACA	
SlCAD^{HaMP}-F	GGCGCGAGCTCTGTATATCCTCGCCGGTATGC	SlCAD^{HaMP}-GFP
SlCAD^{HaMP}-R	TGCGGTCCGATATCATGTAGATTATAACTTTT	
SlCAD^{HaTM}-F	CATTGTGTTCTTTGTTAGGAACCGAACGTTGAATCGCCGCATCG	SlCAD^{HaTM}-GFP
SlCAD^{HaTM}-R	CTACCGCCGCCAGCGCGTAAACCGCCAGGGCGTAATCTCCGCCG	
SlCAD^{HaCPD}-F	GCGAACACGCAGTTCAGAAGAGGAGGCGGTGGCGG	SlCAD^{HaCPD}-GFP
SlCAD^{HaCPD}-R	TTAGTGTCCTAGTCCTGATGAAGAAAGCGATG	
SlCAD$^{HaCR-TB}$-F	GTGAACTCCCAGTTTAAAAGTGGAGGCGGTGGCGG	SlCAD$^{HaCR-TB}$-GFP
SlCAD$^{HaCR-TB}$-R	CTCTGACACGTGTCGATATCAGGTACACCTTCACTTCCGTGCGG	
SlCAD$^{HaTB-MP}$-F	GAAGGTGTACCTGATATCGACACGTGTCAGAG	SlCAD$^{HaTB-MP}$-GFP
SlCAD$^{HaTB-MP}$-R	TGCGGTCCGATATCATGTAGATTATAACTTTT	
SlCAD$^{HaTB-TM}$-F(SlCAD^{HaTM} was used as a background）	GAAGGTGTACCTGATATCGACACGTGTCAGAG	SlCAD$^{HaTB-TM}$-GFP
SlCAD$^{HaTB-TM}$-R	TAGCACGAATCGTCAACTCAAACTGTTGGACA	
SlCAD$^{HaTB-CPD}$-F(SlCAD^{HaCPD} was used as a background）	GAAGGTGTACCTGATATCGACACGTGTCAGAG	SlCAD$^{HaTB-CPD}$-GFP
SlCAD$^{HaTB-CPD}$-R	TAGCACGAATCGTCAACTCAAACTGTTGGACA	

Table S12. Sequence of primers used for amplification of short inserted fragments of *HevCAD*.

Primer	Sequence (5′-3′)	Expressed protein
SlCAD$^{HevTB-MP-TM-CPD}$-F	CCAACAGTTTGAGTTGGTTATTCGTGCTACGGATGGTGGTACGG	SlCAD$^{HevTB-MP-TM-CPD}$-GFP
SlCAD$^{HevTB-MP-TM-CPD}$-R	CACCTCCGCCACCGCCTCCTCTCCTGAGCTGCGAGTTCGCGAAC	
SlCAD^{HevTB}-F	CCAACAGTTTGAGTTGGTTATTCGTGCTACGGATGGTGGTACGG	SlCAD^{HevTB}-GFP
SlCAD^{HevTB}-R	CTCTGACACGTGTCGATATCAGGTACACCTTCACTTCGGTGCGC	

Table S13. Sequence of primers used for amplification of long vector fragments of *SlCAD*.

Primer	Sequence (5′-3′)	Expressed protein
SlCAD$^{HevTB-MP-TM-CPD}$-F	GAACTCGCAGCTCAGGAGAGGAGGCGGTGGCGGAGGTGATCCAC	SlCAD$^{HevTB-MP-TM-CPD}$-GFP
SlCAD$^{HevTB-MP-TM-CPD}$-R	CACCATCCGTAGCACGAATAACCAACTCAAACTGTTGGACACCA	
SlCAD^{HevTB}-F	CGAAGTGAAGGTGTACCTGATATCGACACGTGTCAGAGTAGCCT	SlCAD^{HevTB}-GFP
SlCAD^{HevTB}-R	CACCATCCGTAGCACGAATAACCAACTCAAACTGTTGGACACCA	

Note: The short inserted fragment and the following long vector fragment were used for gene fusion [2].

Table S14. Sequence of primers used for expression of GST tagged CAD fragments in *E.coli* bacteria.

Primer	Sequence (5′-3′)	Expressed protein
GST-TB-MP^{HaCAD}-F	CCGGAATTCTAGGAGGCGGTGGAGGCGGTACGATTCGTGCTACGGACG	GST-TB-MP^{HaCAD}
GST-TB-MP^{HaCAD}-R	CCCAAGCTTCTACAGAGCTCGCGCCTGCGCG	

GST-TB-MP^{SlCAD}-F	CCGGAATTCTAGGAGGCGGTGGAGGCGGTGTTATTCGTGCCACGGATGGT	GST-TB-MP^{SlCAD}
GST-TB-MP^{SlCAD}-R	CCCAAGCTTCTAAACCGCCAGGGCGTAATCT	
GST-CR7-9^{HaCAD}-F	TGCTCTAGACGGAGGCGGTGGAGGCGGTAAGTTCGAGGCGACGGTGTA	GST-CR-7-9^{HaCAD}
GST-CR7-9^{HaCAD}-R	CCGCTCGAGCTACACCTGGAACTCTCGGAAGTCTT	
GST-TB^{HaCAD}-F	CCCAAGCTTAATTCATCGTGACTGACTGACGAT	GST-TB^{HaCAD}
GST-TB^{HaCAD}-R	CCCAAGCTTTCACAGGTACACCTTCACTTCCGT	
GST-MP^{HaCAD}-F	CCGGAATTCTAGGAGGCGGTGGAGGCGGTATATCGGACCGCAACCGAGTGT	GST-MP^{HaCAD}
GST-MP^{HaCAD}-R	CCCAAGCTTCTACAGAGCTCGCGCCTGCGCG	

Table S15. Sequences of the primers used for real time qPCR analyzes.

primers	sequence (5´-3´)
HaCAD-F	CTACGCCATCACAGGTCTTAC
HaCAD-R	CCCCAATAGCCTCTCAAATCC
HaABCC2-F	TTTTGGGCGACTTTGGTGAT
HaABCC2-R	TTCCGTGGGTAGTTTGGTGT
TnCAD-F	TGCTGGACAGAGACGGCGACTAT
TnCAD-R	GTCGTTCACATCTAACAGAACCACG
TnABCC2-F	CACTACACTAGCGACACGGTGCT
TnABCC2-R	GTGCTGACCATAAGGACGGATCTC
Tnrps3A-F	GCGAAAATTCGTCGGAGACGGAG
Tnrps3A-R	ATAATGATGATCTCCGAGCGAGTC

References

1. Xiao, Y.; Dai, Q.; Hu, R.; Pacheco, S.; Yang, Y.; Liang, G.; Soberón, M.; Bravo, A.; Liu, K.; Wu, K. A single point mutation resulting in cadherin mislocalization underpins resistance against *Bacillus thuringiensis* toxin in cotton bollworm. *J. Biol. Chem.* **2017**, 292, 2933–2943.
2. Liu, L.; Chen, Z.; Yang, Y.; Xiao, Y.; Liu, C.; Ma, Y.; Soberón, M.; Bravo, A.; Yang, Y.; Liu, K. A single amino acid polymorphism in ABCC2 loop 1 is responsible for differential toxicity of *Bacillus thuringiensis* Cry1Ac toxin in different S*podoptera* (Noctuidae) species. *Insect Biochem. Mol. Biol.* **2018**, 100, 59–65.
